# Black ginseng under forest as a natural antidepressant: insights into its active components and mechanisms

**DOI:** 10.3389/fchem.2025.1619060

**Published:** 2025-05-30

**Authors:** Yixuan Sui, Yiying Tan, Yajing Li, Xiaochen Gao, Han Lu, Jiaming Shen, Xuesheng Hu, Lei Wang, Liting Zhao, Jiaming Sun, Chunnan Li

**Affiliations:** ^1^ Jilin Ginseng Academy, Changchun University of Chinese Medicine, Changchun, China; ^2^ School of Pharmacy, Changchun University of Chinese Medicine, Changchun, China

**Keywords:** black ginseng under forest (BG), UPLC-QE Orbitrap-MS, depression, neuroinflammation, ginsenoside F1

## Abstract

Depression is a psychological disorder with significant global impact. It is widely hypothesized that this disorder is associated with neuroinflammation, which disrupts neural homeostasis through various pathways. This study aims to investigate the effective compounds and mechanisms of Black Ginseng under forest (BG) in combating neuroinflammation. Utilizing methods such as UPLC-QE Orbitrap-MS, network pharmacology, molecular docking, and cell biology, the efficacy of BG was demonstrated, and its active components were identified. Cell viability and apoptosis were assessed using Trans well migration assays and flow cytometry. The mRNA expression of target genes was confirmed through real-time quantitative PCR (RT-qPCR), elucidating the anti-neuroinflammatory mechanism. The results indicated that BG exhibited a more pronounced effect on ameliorating neuroinflammatory conditions compared to Ginseng under forest (FG). The main active components were found through research and development, including Ginsenoside F1, Ginsenoside Rk1, Ginsenoside Rg3, etc. Among these, Ginsenoside F1 emerged as the most potent active component for treating neuroinflammation, as evidenced by reduced cell migration and apoptosis. The study demonstrates that BG can modulate the PI3K-Akt signaling pathway, leading to a reduction in the expression levels of AKT1, MAPK1, PIK3CA, EGFR, and other mRNAs. These findings suggest that BG is a promising natural antidepressant, providing both theoretical and experimental foundations for the development of new antidepressants based on BG and its active components.

## 1 Introduction

Major depressive disorder (MDD), also referred to as depressive disorder, is the most prevalent mental illness (Monroe and Harkness, 2022). Clinically, it manifests as a common emotional mental disorder characterized by symptoms such as low mood, irritability, inattention, pessimism, and cognitive and sleep disturbances (Wang et al., 2015). The pathogenesis of MDD is complex, with a wide range of symptoms, making it difficult to treat. It is often associated with various comorbidities, including diabetes (Campayo et al., 2011), hypertension, coronary heart disease (Zhang et al., 2018), and epilepsy (Błaszczyk and Czuczwar, 2016). Consequently, depression has emerged as one of the most significant and severe mental disorders globally, exerting substantial adverse effects on individuals, families, and society. Currently, the pharmacological treatment of depression in clinical settings is predominantly categorized into five groups: tricyclic antidepressants, monoamine oxidase inhibitors (Li et al., 2024), selective serotonin reuptake inhibitors (SSRIs) (Nutt, 1999), atypical antidepressants, and other miscellaneous antidepressants. Nevertheless, the majority of these pharmacotherapies are associated with numerous adverse reactions and potential toxic side effects. Additional challenges include delayed therapeutic onset, development of drug resistance, high relapse rates, and persistent residual symptoms (Paschos et al., 2009). Consequently, there is an urgent demand for safe, side-effect-free, and efficacious natural medicines or health supplements for the treatment of depression.

Ginseng (Panax ginseng C. A. Mey.) is divided into Garden ginseng (GG) and ginseng under forest (FG). GG is an artificially cultivated ginseng with the age of about 5 years, whereas FG is a plant that grows naturally in mountain forests for 10–20 years after artificial seeding, according to the Chinese Pharmacopoeia (Hao et al., 2022). Thus, identifying natural and effective therapeutic agents devoid of adverse effects is of paramount importance. In this context, FG is recognized as a traditional Chinese medicine with diverse pharmacological properties (Liu et al., 2020), including modulation of the central nervous system (Lu et al., 2022), hypoglycemic effects (Xie et al., 2005), and antioxidant activities. The primary bioactive constituents of ginseng include ginsenosides, ginseng polysaccharides, and ginseng peptides, with saponins being identified as the principal active components (Shin et al., 2015). Existing literature suggests that ginseng may possess therapeutic potential for the treatment of depression and neuroinflammation (Jin et al., 2019). Black ginseng under forest (BG) is derived from the dried root and rhizome of ginseng, which undergoes multiple steaming and drying processes, resulting in a novel processed ginseng product. Initial investigations revealed a substantial increase in the concentration of rare saponins in BG post-processing. Additionally, other secondary metabolites, such as phenolic compounds, reducing sugars, and acidic polysaccharides, also exhibited significant increases (Metwaly et al., 2019). These compounds demonstrated enhanced pharmacological activities in the treatment of hypercholesterolemia (Saba et al., 2016) and aging compared to traditional ginseng (Lee et al., 2022). It is worth noting that chemical compounds bearing alcoholic groups, especially phenol-based compounds, are considered as multifunctional and important agents in pharmaceutical development and medicinal chemistry (Klika et al., 2021; Makarem et al., 2023; Makarem et al., 2019). Currently, research on the antidepressant effects of BG is limited, and it remains unclear whether its efficacy in alleviating depression and neuroinflammation surpasses that of conventional ginseng. Furthermore, the active components and mechanisms underlying these effects are not well understood.

To address these gaps, this study employs UPLC-QE Orbitrap-MS technology in conjunction with network pharmacology, molecular docking, cell experiments, and RT-qPCR techniques. BG serves as the focal point of this research, with an analysis of the differences in pharmacological efficacy pre- and post-processing. The study aims to identify the most potent active components and validate their anti-neuroinflammatory effects. In conclusion, the pharmacological compounds and mechanisms by which BG alleviates neuroinflammation have been elucidated, demonstrating its antidepressant effects. This provides both theoretical and experimental foundations for the development of novel antidepressant agents.

## 2 Materials and methods

### 2.1 Materials and reagents

FG sample (batch number: TF 0321) was purchased from China Changchun Hong Jian Pharmacy. The material was identified by Professor Sun Jiaming from Changchun University of Traditional Chinese Medicine in China, and it met the quality standards outlined in China Pharmacopoeia (2020 edition). Chromatographic acetonitrile (LOT NO. L-3301) was purchased from Fisher Scientific, chromatographic formic acid (LOT NO. F809712) was purchased from Shanghai McLean Biochemical Technology Co., Ltd., and purified water (LOT NO. 17323) was purchased from Hangzhou Wahaha Group Co., Ltd. 4% paraformaldehyde (LOT NO. 24101933) was purchased from Bio sharp Company (Guang dong, China); Lipopolysaccharide (LPS, LOT NO. S11060), Ginsenoside Rk1(CAS: 494753-69-4, HPLC≥98%), Ginsenoside Rg3 (CAS: 14197-60-5, HPLC≥98%), 20 (r)-Ginsenoside Rh1 (CAS: 80952-71-2, HPLC≥98%), 20 (s)-Ginsenoside Rh1 (CAS: 63223-86-9, HPLC≥98%), Ginsenoside F1. 5-hydroxytryptamine (5-HT, LOT NO. ml365221-J), dopamine (DA, LOT NO. ml002024), interleukin −6(Interleukin −6, IL-6, LOT NO. ml063159), tumor necrosis factor-α (TNF-α, LOT NO. ml002095), and the ELISA detection kit was purchased from Shanghai enzyme-linked biotechnology Co. Ltd.

### 2.2 BG sample preparation

The preparation of BG involves taking 10 roots of FG, each aged 15 years, and pulverizing them into a coarse powder. The powder is then placed in a steaming container wrapped tightly with gauze and subjected to a high-pressure steam sterilization process at 120°C, followed by stewing. After steaming and stewing, the material is dried in an oven at 60°C for 8 h. This process is repeated nine times to produce BG. For extraction, 1 g samples of FG and BG are weighed and placed in separate conical flasks, to which 20 mL of a 70% methanol solution is added. The samples are subjected to ultrasonic extraction for 1 h, followed by centrifugation to obtain the supernatant, leaving the residue behind. This extraction process is repeated twice, and the filtrates are combined. The solvent is then recovered under reduced pressure to yield the 70% methanol extracts of the two samples.

### 2.3 UPLC-QE Orbitrap-MS mass spectrometry analysis of chemical components in BG

The chemical components in BG were analyzed by ultra performance liquid chromatography-quadrupole-Orbitrap mass spectrometry (UPLC-QE Orbitrap-MS). SB-C18 column (4.6 × 150 mm; Agilent, United States), column temperature: 30°C, flow rate: 0.3 mL/min. The mobile phase is a: 0.1% formic acid; B: 100% acetonitrile, sample injection amount: 10 μL. The gradient elution procedure is: 0–20 min, 15%–36% B; 20–25 min, 36% B; 25–32 min, 36%–41% B; 32–42 min, 41%–60% B; 42–52 min, 60%–100% B; 52–62 min, 100% B; 62–65 min, 100%–15% B; 65–75 min, 15% B. Electrospray ion source (ESI), negative ion mode detection, dry gas temperature: 350°C, sheath gas flow rate: 4 × 10^6^ Pa, auxiliary gas flow rate: 1 × 10^6^ Pa, auxiliary gas temperature: 300°C, scanning mode: full scanning -ddMS2, mass scanning range: 150–2,000 m/z. Use Xcalibur software to collect and analyze data.

### 2.4 A comparative analysis of the efficacy of FG and BG is conducted *in vitro*

#### 2.4.1 Cell culture

HT22 cells were procured from the Shanghai Chinese Academy of Sciences, China, and their proliferation was monitored using an optical microscope. The cells were cultured in a 5% CO_2_ incubator at 37°C, with the culture medium consisting of DMEM supplemented with 10% fetal bovine serum and 1% penicillin (100 U/mL)-streptomycin (100 μg/mL). The culture medium was refreshed every 2 days. Once the cells reached 90% confluence, they were subcultured at a 1:2 ratio, and the experiments commenced when the cells entered the logarithmic growth phase.

#### 2.4.2 Establishment method of neuroinflammation model

Logarithmically growing HT22 cells were seeded into 96-well plates at a density of 5 × 10^3^ cells per well, with PBS added to the peripheral wells to mitigate edge effects. The cells were divided into a Control group and Lipopolysaccharide (LPS) treatment groups with varying concentrations (1, 2, 4, 8, 16 μg/mL). Following a 24-h incubation period, 100 μL of culture medium and 10 μL of CCK-8 solution were added to each well. After a 30-min incubation, the optical density (OD) was measured at 450 nm to calculate cell viability. The neuroinflammation model was deemed successfully established when cell damage reached approximately 30%, and the LPS concentration at this point was designated as the modeling concentration.

Cell survival % = OD value of experimental group/OD value of blank group × 100%.

#### 2.4.3 Experiment and grouping method of FG and BG on LPS-induced neuroinflammation model of HT22 cells

The procedures for paving and molding were consistent with those outlined in Section 2.4.2. The experimental groups included a control group, a model group, and FG and BG groups with concentrations of 100, 50, 25, 12.5, and 6.25 μg/mL. Following administration and a 24-h incubation period, the concentrations of inflammatory markers TNF-α and IL-6, as well as monoamine neurotransmitters DA and 5-HT, were quantified in the cell supernatant using an ELISA kit, and cell viability was assessed.

#### 2.4.4 Screening of ginsenosides from HT22 neurons induced by LPS

For the screening of ginsenosides in HT22 neurons induced by LPS, the experimental groups consisted of a control group, a model group, and a ginsenoside group with concentrations of 50, 25, 12.5, and 6.25 μM. After 24 h of incubation, the concentrations of TNF-α and IL-6, along with DA and 5-HT, were measured in the cell supernatant using the ELISA kit, and cell viability was evaluated.

### 2.5 Network pharmacological analysis

#### 2.5.1 Screening and collection of active ingredients

The chemical constituents analyzed using UPLC-QE Orbitrap-MS were identified in the TCMSP database (http://tcmspw.com/tcmsp.php/) based on the following screening criteria: oral bioavailability (OB) ≥ 30%, blood-brain barrier permeability (BBB) ≥ 0.3, and drug-likeness (DL) ≥ 0.1. Additional information was supplemented through a review of the literature. The SMILES notation for each component was obtained from the PubChem database (https://pubchem.ncbi.nlm.nih.gov/) and subsequently analyzed using the Swiss ADME database (http://www.swissadme.ch/). The effective active components were selected based on criteria of high gastrointestinal absorption, BBB permeability, and a Lipinski score >0.5.

#### 2.5.2 Predict the corresponding targets of components in BG

After obtaining the SMILES number of the active ingredient in 2.5.1 on the PubChem website (https://PubChem.ncbi.nlm.nih.gov/), the target corresponding to the ingredient was searched by Swiss Target Prediction (http://www.swisstargetprediction.ch/), and the obtained information was searched with the BATMAN-TCM database for potential activity.

#### 2.5.3 Acquisition of depression-related targets

Using “Neuroinflammation” as a keyword, targets associated with depression-related diseases were identified through the Gene Cards (https://www.genecards.org), DisGeNET (https://www.disgenet.org/), and OMIM (https://www.omim.org) databases. In the Gene Cards database, disease target proteins were further filtered to include only those with a Relevance score exceeding the average. Similarly, in the DisGeNET database, targets were retained if their Score value exceeded the median, while those with lower scores were excluded. Download the disease-associated targets from the Gene Map within the OMIM database and integrate these targets with those obtained from the other two databases to compile comprehensive target information pertaining to neuroinflammation.

#### 2.5.4 Acquisition of potential targets of intersection

Utilizing the Venny 2.1.0 platform, the targets corresponding to potential active ingredients identified in Sections 2.5.2 and 2.5.3 were amalgamated with the disease-related target information. A Venn diagram was constructed to identify common targets, which were subsequently extracted as potential targets for BG intervention in depression.

#### 2.5.5 Enrichment analysis of go and KEGG pathways

The intersection of disease targets and potential targets was introduced into the DAVID database for gene ontology (GO) and Kyoto Encyclopedia of Genes and Genomes (KEGG) pathway enrichment analysis. This analysis aims to elucidate the biological processes and signaling pathways potentially involved in the pharmacological treatment of neuroinflammation. The enrichment analysis data were visualized using the -log10 P-value as the ordinate on the micro-information platform.

#### 2.5.6 BG “drug-potential active ingredient-target-pathway” network diagram construction

The top 20 pathways identified through KEGG enrichment analysis of active drug components, core targets, and associated targets were imported into Cytoscape 3.10.2 software to construct a network diagram illustrating the active components, core targets, and pathways involved in the treatment of neuroinflammation by BG. Topological analysis was conducted using the CytoNCA plugin, allowing for the identification of potential key active components, targets, and pathways based on topological parameters such as node Degree (DC) and Betweenness (BC).

### 2.6 Molecular docking and molecular dynamics simulation verify the interaction between active components and core targets

#### 2.6.1 Molecular docking analysis

Molecular docking was performed using Auto Dock, with 3D coordinate files of the active components obtained from the PubChem database (https://pubchem.ncbi.nlm.nih.gov/) and 3D files of the disease core targets retrieved from the PDB database (https://www.rcsb.org). The spatial structures were verified using PyMOL. Sail Vina was employed to convert the docking ligands into pdbqt format, and Auto Dock Tools 1.5.6 was utilized to remove water molecules from the protein complexes, with the ligands and receptors separated and stored as pdbqt files for docking purposes. Finally, PyMOL software was used to generate 3D interactive visualization.

#### 2.6.2 Molecular dynamics simulation analysis

The initial configuration for the molecular dynamics simulation was derived from molecular docking results. The simulation was conducted using the AMBER99SB force field within the GROMACS 2023.2 software environment. A water box was established, employing the TIP3P water model, and normal saline was simulated by adding NaCl ions to achieve charge neutrality. Energy minimization was performed using the steepest descent method, followed by system equilibration for 1 ns at 300 K and 1 ns under atmospheric pressure. A 50 ns molecular dynamics simulation was subsequently executed. Analyses of the simulation system included root mean square deviation (RMSD), root mean square fluctuation (RMSF), radius of gyration (RG), and hydrogen bonding.

### 2.7 RT-PCR analysis

Groups were categorized as follows: Control: Complete medium; LPS group: 2 μg/mL LPS; BG group, which included a high dose of 100 μg/mL and a low dose of 50 μg/mL, and the Ginsenoside F1 group, with a high dose of 12.5 μM and a low dose of 6.25 μM.

Total RNA was extracted from HT22 cells, and absorbance values at 260 and 280 nm were measured to determine RNA purity and concentration. The mRNA expression levels of epidermal growth factor receptor (EGFR), phosphoinositide 3-kinase catalytic subunit α (PIK3CA), protein kinase B (AKT1), and mitogen-activated protein kinase (MAPK1) within the PI3K-Akt signaling pathway were quantified using quantitative reverse transcription PCR (qRT-PCR). Based on the known concentration measurements, the necessary RNA content was calculated, and genomic DNA removal, reverse transcription, and PCR amplification reactions were performed. GAPDH served as the internal reference for gene normalization, and the 2^−ΔΔCT^ method was employed for data analysis. The primer sequences for the upstream and downstream regions of the relevant target proteins are detailed in Table 1.

**TABLE 1:** Primers sequence.

Target	Forward primer	Reverse primer
GAPDH	CTG​GAG​AAA​CCT​GCC​AAG​TAT​G	GGTGGAAGAATGGG AGTTGCT
EGFR	AGT​CGG​GCT​CTG​GAG​GAA​AA	CCA​AGG​ACC​ACC​TCA​CAG​TTA​T
PIK3CA	CCA​CGA​CCA​TCA​TCA​GGT​GAA	CCT​CAC​GGA​GGC​ATT​CTA​AAG​T
AKT1	TGC​ACA​AAC​GAG​GGG​AGT​ACA	GCG​CCA​CAG​AGA​AGT​TGT​TGA
MAPK1	TAC​ACC​AAC​CTC​TCG​TAC​ATC​G	CAT​GTC​TGA​AGC​GCA​GTA​AGA​TT

### 2.8 Apoptosis was detected by flow cytometry

Following a 24-h incubation period, HT22 cells were collected, digested using trypsin without EDTA, centrifuged, and washed twice with precooled PBS. Subsequently, 5 μL of Annexin V-FITC and 5 μL of PI were added, and the mixture was incubated in the dark for 15 min. Apoptosis rates were determined via flow cytometry and analyzed using FlowJo_v10.8.1_CL software. The grouping method is consistent with item 2.7.

### 2.9 Trans well detection of cell migration

For the migration assay, a 24-well plate with an 8 μM Trans well chamber was utilized, and 5 × 10^4^ HT22 cells were seeded into the upper chamber. In the control group, 600 μL of complete medium containing FBS was added to the lower chamber. For the experimental group, 600 μL of FBS with drug medium was cultured for 24 h. The lower chamber was washed twice with PBS and fixed with paraformaldehyde at room temperature for 15 min. Crystal violet staining was performed for 15 min, followed by multiple PBS washes until the background was clear. Images were captured under a 10× microscope and quantified using ImageJ software. The grouping method is consistent with item 2.7.

### 2.10 Statistical analysis

Statistical analyses were performed using the statistical software Python (Python 2.7.6 version), with graphing using GraphPad Prism 8.0 (GraphPad Software, San Diego, CA, United States). The data were expressed in terms of mean ± standard deviation (SD). A p-value <0.01 was deemed to be statistically significant.

## 3 Results

### 3.1 Effect of LPS on the survival rate of neurons (HT22)

**FIGURE 1:**
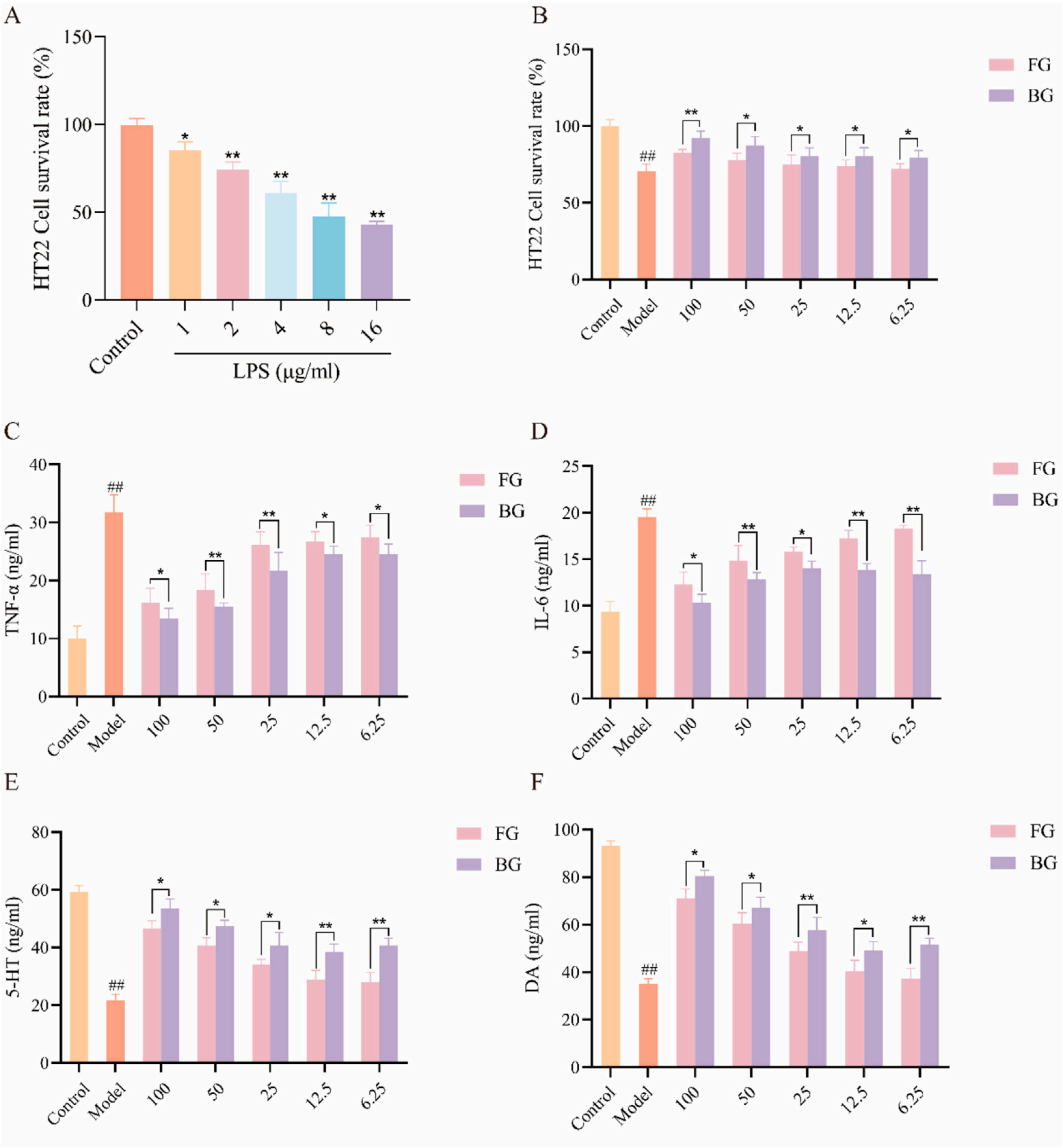
Pharmacodynamic analysis of FG and BG. **(A)** Model of HT22 Neuroinflammation Induced by LPS; **(B)** HT22 cell activity; **(C)** Inflammatory factor TNF-α; **(D)** Inflammatory factor IL-6; **(E)** Monoamine neurotransmitter 5-HT; **(F)** Monoamine neurotransmitter DA (data expressed as mean ± SD [*n* = 3], ^##^ *P* < 0.01 compared to the control group, *P < 0.05, **P < 0.01 compared to the model group).

HT22 cells were exposed to varying concentrations of lipopolysaccharide (LPS) for a duration of 24 h. The results obtained from the CCK-8 assay (Figure 1A) indicated that increasing concentrations of LPS led to a reduction in cell viability. Specifically, at an LPS concentration of 2 μg/mL, approximately 30% damage to HT22 cells was observed, which was deemed successful for modeling purposes. Consequently, subsequent experiments were conducted using this concentration.

### 3.2 Effects of FG and BG on survival rate and related factors of HT22 cells induced by LSP

The findings (Figure 1B) demonstrate that the cell survival rate in the BG group was higher than that in the FG group at equivalent doses. Additionally, the levels of TNF-α (Figure 1C) and IL-6 (Figure 1D) were assessed, revealing that BG more effectively reduced the expression of these inflammatory markers and mitigated the neuroinflammatory response. Furthermore, BG exhibited a notable impact on the secretion of monoamine neurotransmitters, dopamine (DA), and serotonin (5-HT) (Figures 1E,F).

### 3.3 UPLC-QE Orbitrap-MS analysis

**FIGURE 2:**
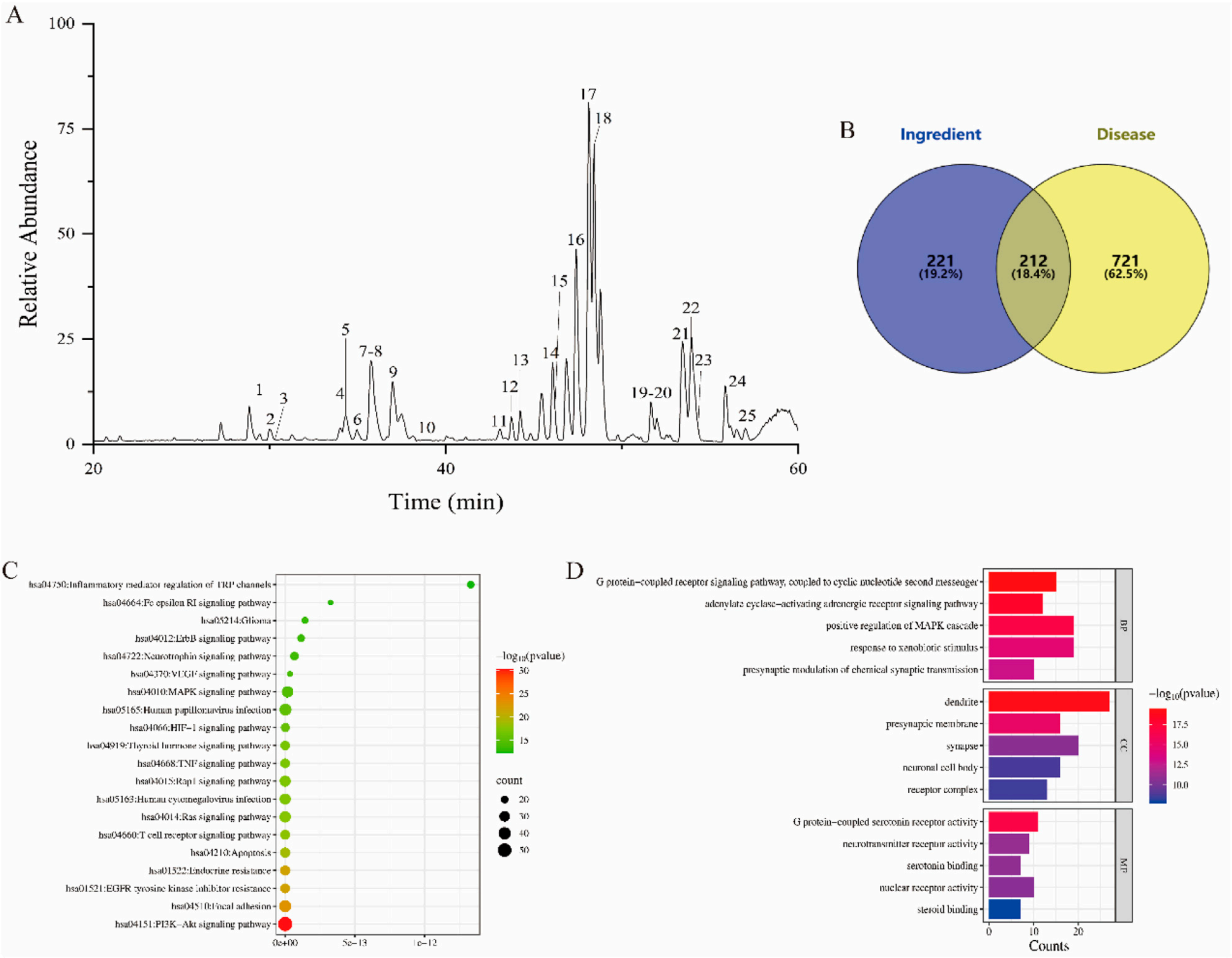
BG component analysis. **(A)** Total ion current chromatogram in negative ion mode. **(B)** Wayne diagram of intersection target; **(C)** KEGG analysis; **(D)** Go analysis.

The chemical composition of BG was analyzed using UPLC-QE Orbitrap-MS, with the total ion chromatogram (ESI-) presented in Figure 2A. Compounds were tentatively identified by comparing molecular ion peaks, fragment ion information, and retention times with existing databases or literature reports, resulting in the identification of 25 chemical constituents. Refer to Table 2 for detailed information.

**TABLE 2:** Chemical composition analysis results of BG based on UPLC-QE-Orbitrap-MS technology.

No.	Time	Chemical compound	Formula	Adducts	Measured weight	MS_2_ characteristic ions (m/z)
1	29.90	Ginsenoside Rg1	C_42_H_72_O_14_	[M + COOH]-	845.4947	799.4941, 637.4355, 475.3810
2	30.00	Ginsenoside Rf	C_42_H_72_O_14_	[M + COOH]-	845.4947	799.4893, 637.4351, 475.3828
3	30.19	Ginsenoside Rb_1_	C_54_H_92_O_23_	[M-H]-	1107.5980	1107.6006, 945.5461, 783.4932
4	33.92	Ginsenoside Ro	C_48_H_76_O_19_	[M-H]-	955.4947	955.4944, 793.4429
5	34.33	20(S)-Ginsenoside Rg2	C_42_H_72_O_13_	[M-H]-	829.4985	783.4937, 637.4343, 475.3817, 391.2873
6	34.95	20(R)-Ginsenoside Rg2	C_42_H_72_O_13_	[M-H]-	829.4985	783.493, 637.4351, 475.3824
7	35.72	20(S)-Ginsenoside Rh1	C_36_H_62_O_9_	[M + COOH]-	683.4404	637.4338, 475.3790, 161.0462
8	35.72	20(R)-Ginsenoside Rh1	C_36_H_62_O_9_	[M + COOH]-	683.4404	637.4363, 475.3806
9	37.01	Ginsenoside F1	C_36_H_62_O_9_	[M-H]-	683.4404	637.4363
10	38.69	Ginsenoside Rd	C_48_H_80_O_18_	[M + COOH]-	991.4509	945.4463, 783.4927, 621.4355, 459.3810
11	43.02	Ginsenoside Rg8	C_42_H_70_O_13_	[M + COOH]-	827.4839	781.4781, 619.4238, 221.0681, 161.0457
12	43.69	Ginsenoside Rg9	C_42_H_70_O_13_	[M + COOH]-	827.4839	781.4775, 619.4237, 221.0679, 161.0458
13	44.36	Ginsenoside F2	C_42_H_72_O_13_	[M + COOH]-	829.4985	783.4930, 621.4412
14	46.10	Ginsenoside F4	C_42_H_70_O_12_	[M-H]-	811.4883	783.4802, 603.4296
15	46.19	Ginsenoside Rk1	C_42_H_70_O_12_	[M + COOH]-	811.4883	765.4833,619.4240
16	47.41	Ginsenoside Rk3	C_36_H_60_O_8_	[M + COOH]-	665.4303	619.4213, 161.0458, 101.0239
17	48.14	Ginsenoside Rh4	C_36_H_60_O_8_	[M + COOH]-	665.4303	619.4254,161.0455,101.0239
18	48.42	Ginsenoside Rg3	C_42_H_72_O_13_	[M + COOH]-	829.4985	783.4937, 621.4401, 221.0679, 161.0458
19	51.64	20(S)-Ginsenoside Rs3	C_42_H_70_O_12_	[M + COOH]-	871.5101	825.5037, 783.4940, 621.4411, 459.3862, 161.0459
20	51.64	20(R)-Ginsenoside Rs3	C_44_H_74_O_14_	[M + COOH]-	871.5101	783.4941, 621.4402, 459.3857, 161.0459
21	53.46	Ginsenoside Rg4	C_42_H_70_O_12_	[M + COOH]-	811.4883	765.4829
22	53.94	Ginsenoside Rg5	C_42_H_70_O_12_	[M + COOH]-	811.4883	765.4824, 211.0680
23	54.38	Ginsenoside Rg6	C_42_H_70_O_12_	[M-H]-	811.4883	603.4305
24	56.52	Ginsenoside RS4	C_44_H_72_O_13_	[M + COOH]-	853.4996	807.4963, 765.4834, 603.4278, 161.0458
25	57.03	Ginsenoside RS5	C_44_H_72_O_13_	[M + COOH]-	853.4995	765.4827, 603.4269

### 3.4 Network pharmacological analysis results

#### 3.4.1 Corresponding targets of chemical components in BG

The study identified the corresponding targets of 25 chemical components analyzed using UPLC-QE-Orbitrap-MS, resulting in the retrieval of 433 targets.

#### 3.4.2 Related targets of depression

By employing “Neuroinflammation” as a keyword, searches were conducted in databases such as Gene Cards and OMIM, yielding 933 targets related to depression after merging and removing duplicates.

#### 3.4.3 The intersection target of BG and depression

A Venn diagram was constructed to compare the corresponding targets of BG with those related to neuroinflammation, as outlined in Section 3.4.1, resulting in 212 intersecting targets. The Venn diagram was generated using Venny 2.1.0, as depicted in Figure 2B.

#### 3.4.4 Analysis of GO and KEGG pathways of targets related to BG therapy for depression

Comprehensive analysis of KEGG pathway enrichment results (Figure 2C) indicates that the primary pathways associated with BG include the VEGF signaling pathway, HIF-1 signaling pathway, Ras signaling pathway, and neuroactive ligand-receptor interaction pathway. The GO analysis results for BG (Figure 2D) reveal that the biological processes involved in its therapeutic effects on neuroinflammation include the positive regulation of the MAPK1 cascade and the response to exogenous stimuli, which are closely linked to the mechanism underlying BG’s therapeutic efficacy in treating neuroinflammation. Molecular functions encompass G protein-coupled serotonin receptor activity, serotonin binding activity, and neurotransmitter receptor activity.

#### 3.4.5 BG’s “Drug-Component-Target-Pathway” network diagram

**FIGURE 3:**
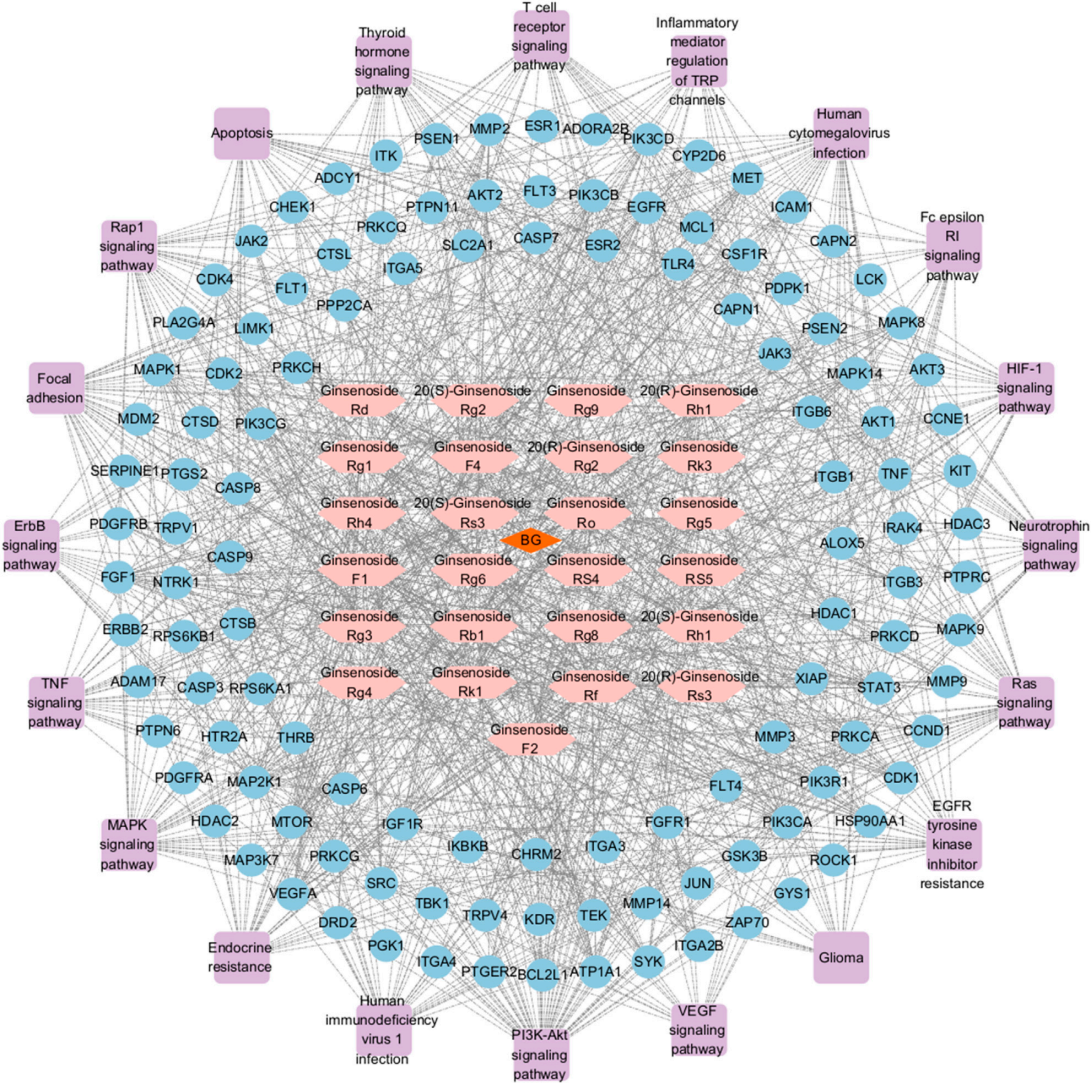
BG’s “Drug-Component-Target-Pathway” Network Diagram (Pink is the chemical component in BG; Blue is the core target; Purple is the key pathway).

The network of BG comprises 166 nodes, which include 120 core targets, 25 potential active components of BG, and 20 pathways. Refer to Figure 3 for a depiction of the 1,197 connections. Post-analysis, 45 nodes exceeding the average of the three calculated values were identified, as detailed in Table 3. Through topological analysis of the network diagram—where the node degree, betweenness, and closeness centrality values of each component were ranked from highest to lowest—it was determined that the core components involved in neuroinflammation intervention are Ginsenoside Rk1 and Ginsenoside Rg3. The key core targets identified include PIK3CA and AKT1.

**TABLE 3:** BG “Drug-Component-Target-Pathway” network node information.

No.	Name	Degree	CC	BC	Source
1	PI3K-Akt signaling pathway	56	0.467605634	0.088947162	Pathway
2	Ginsenoside Rk1	41	0.447439353	0.03284333	Component
3	Ginsenoside Rg3	41	0.447439353	0.03284333	Component
4	20(S)-Ginsenoside Rh1	39	0.442666667	0.042331619	Component
5	20(R)-Ginsenoside Rh1	39	0.442666667	0.042331619	Component
6	Focal adhesion	38	0.42455243	0.035785562	Pathway
7	Ginsenoside F1	37	0.437994723	0.032763253	Component
8	Ginsenoside Rg6	36	0.435695538	0.044578208	Component
9	Ras signaling pathway	35	0.41813602	0.024409554	Pathway
10	MAPK signaling pathway	35	0.41813602	0.026980157	Pathway
11	Human cytomegalovirus infection	34	0.4160401	0.025961671	Pathway
12	PIK3CA	33	0.532051282	0.030512759	Target
13	Ginsenoside Ro	33	0.428940568	0.049263521	Component
14	Rap1 signaling pathway	33	0.413965087	0.023261252	Pathway
15	Ginsenoside Rk3	31	0.42455243	0.023938356	Component
16	Ginsenoside F4	31	0.42455243	0.019457523	Component
17	AKT1	30	0.51875	0.023400433	Target
18	Ginsenoside Rg1	30	0.422391858	0.027623287	Component
19	Ginsenoside F2	30	0.422391858	0.032477485	Component
20	Ginsenoside Rg5	30	0.422391858	0.030669602	Component
21	Human immunodeficiency virus 1 infection	30	0.407862408	0.02222399	Pathway
22	Apoptosis	29	0.405867971	0.032007658	Pathway
23	Ginsenoside Rg4	28	0.41813602	0.015838078	Component
24	MAPK1	28	0.509202454	0.020040098	Target
25	Ginsenoside H2	28	0.41813602	0.019667335	Component
26	20(S)-Ginsenoside Rg2	28	0.41813602	0.019001281	Component
27	20(R)-Ginsenoside Rg2	28	0.41813602	0.019001281	Component
28	Endocrine resistance	28	0.403892944	0.019100345	Pathway
29	Ginsenoside Rb1	27	0.4160401	0.018084305	Component
30	Ginsenoside Rd	27	0.4160401	0.016771038	Component
31	MTOR	27	0.512345679	0.018653974	Target
32	T cell receptor signaling pathway	27	0.401937046	0.024286155	Pathway
33	EGFR	26	0.509202454	0.016633884	Target
34	VEGFA	25	0.497005988	0.017242694	Target
35	FGF1	23	0.494047619	0.015755153	Target
36	PIK3CD	23	0.48255814	0.012178646	Target
37	PIK3CB	23	0.477011494	0.013371433	Target
38	PIK3R1	23	0.500000000	0.01313557	Target
39	MAP2K1	22	0.494047619	0.012826497	Target
40	STAT3	21	0.477011494	0.011052891	Target
41	AKT2	21	0.488235294	0.010635294	Target
42	MAPK8	20	0.477011494	0.010701626	Target
43	MAPK14	20	0.468926554	0.011159358	Target
44	AKT3	20	0.485380117	0.009533495	Target
45	PRKCA	19	0.461111111	0.01040643	Target

### 3.5 Screening of active components in HT22 cell neuroinflammation model induced by LPS

**FIGURE 4:**
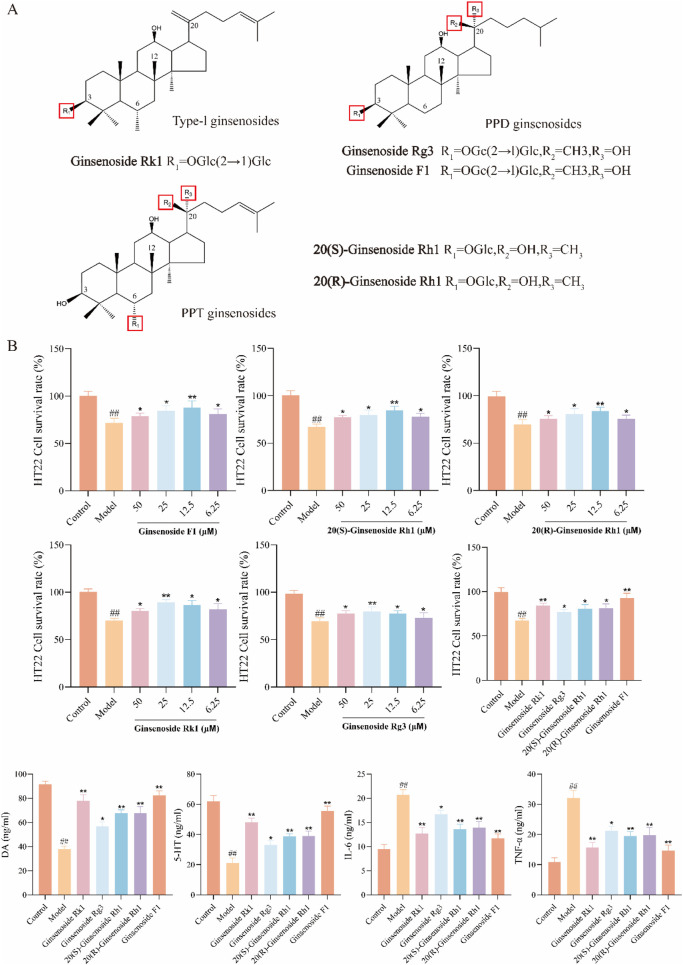
Screening of active ingredients. **(A)** Five compounds screened from BG; **(B)** Screening of Active Components from Neuroinflammation induced by LPS in HT22 Cells (data expressed as mean ± SD [*n* = 3], ^##^ *p* < 0.01 compared to the control group, *p < 0.05, **p < 0.01 compared to the model group).

Based on network pharmacology analysis, the top five active ingredients are Ginsenoside Rk1, Ginsenoside Rg3, 20(S)-Ginsenoside Rh1, 20(R)-Ginsenoside Rh1, and Ginsenoside F1 (Figure 4A). These active components were further validated using an LPS-induced neuroinflammation model in HT22 cells. Figure 4B illustrates that the cell survival rate in the Model group decreased, while cell activity increased following a 24-h incubation with active components. This increase in cell activity is concentration-dependent, with a final drug concentration of 12.5 μM used for comparison. Our results indicate that Ginsenoside F1 exhibited the highest cell survival rate. Concurrently, the levels of TNF-α, IL-6, DA, and 5-HT were quantified using ELISA, leading to the selection of Ginsenoside F1 as the most effective active component for further analysis.

### 3.6 Molecular docking results and molecular docking mode analysis

**FIGURE 5:**
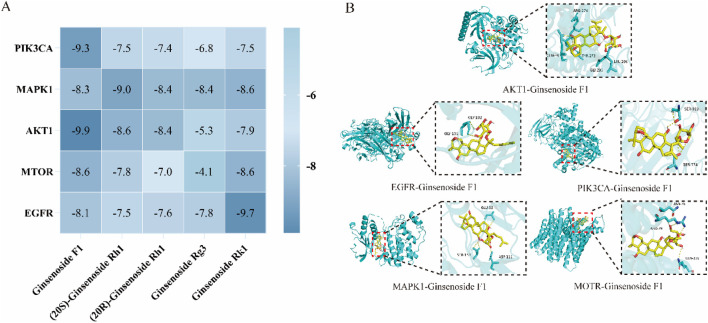
Molecular docking analysis. **(A)** Molecular docking result; **(B)** Visualization of molecular docking.

The top five core targets identified in Section 3.4.5 were subjected to molecular docking with Ginsenoside F1. The results, presented in Figure 5A, demonstrate a close interaction between the active ingredient and the core targets, with a binding energy of −8.9 kcal/mol for the Ginsenoside F1-AKT1 complex, indicating a strong affinity with the core target protein. Additionally, PyMOL software was employed to generate a 3D visualization of the target-ligand interactions (Figure 5B).

### 3.7 Molecular dynamics simulation analysis results

**FIGURE 6:**
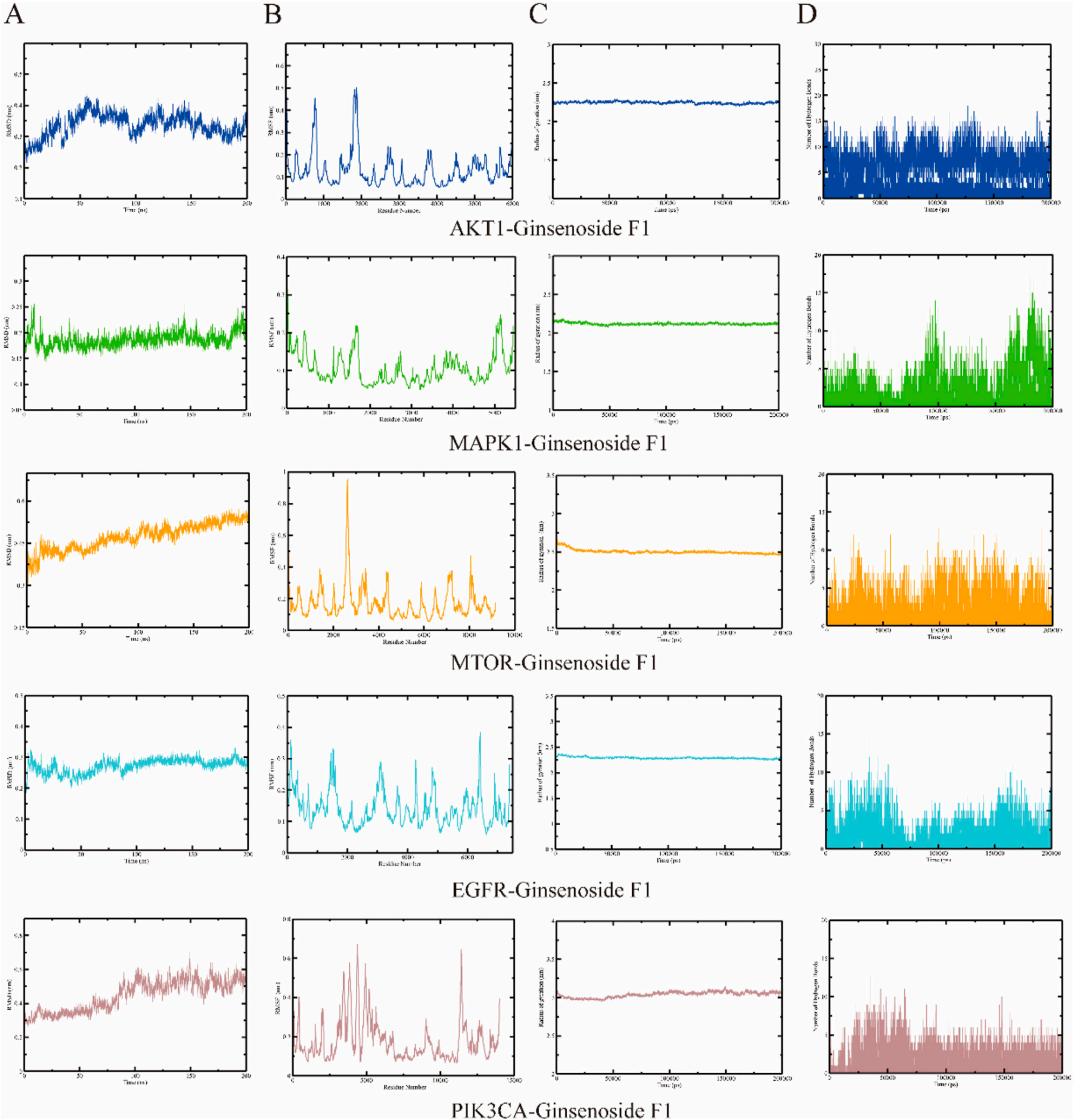
Molecular dynamics simulation analysis. **(A)** RMSD analysis; **(B)** RMSF analysis; **(C)** RG analysis; **(D)** Hydrogen bond analysis.

The root mean square deviation (RMSD) quantifies the cumulative atomic deviations between a given conformation at a specific time point and a reference conformation, serving as a crucial metric for assessing system stability. A consistently low RMSD value indicates a strong affinity between the ligand and receptor. Analysis of the RMSD across five molecular dynamics simulation systems reveals a variation of less than 0.4 nm, suggesting stable binding (Figure 6A). The root mean square fluctuation (RMSF) measures the flexibility of amino acid residues in proteins, providing an evaluation of the root mean square displacement of each residue and reflecting their fluctuations. The results (Figure 6B) demonstrate that the ligand and target amino acid residues exhibit stable binding with minimal fluctuation. The radius of gyration (Rg) is employed to describe changes in the overall structural conformation, where larger Rg variations indicate greater architectural changes. Throughout the simulation process, the Rg remained consistently low, with differences less than 0.1 nm, indicating stable complex formation (Figure 6C). Hydrogen bonds are crucial for the stable existence of coordination compounds. As illustrated in Figure 6D, all five systems achieved a relatively stable state during the simulation process.

### 3.8 qRT-PCR analysis results

**FIGURE 7:**
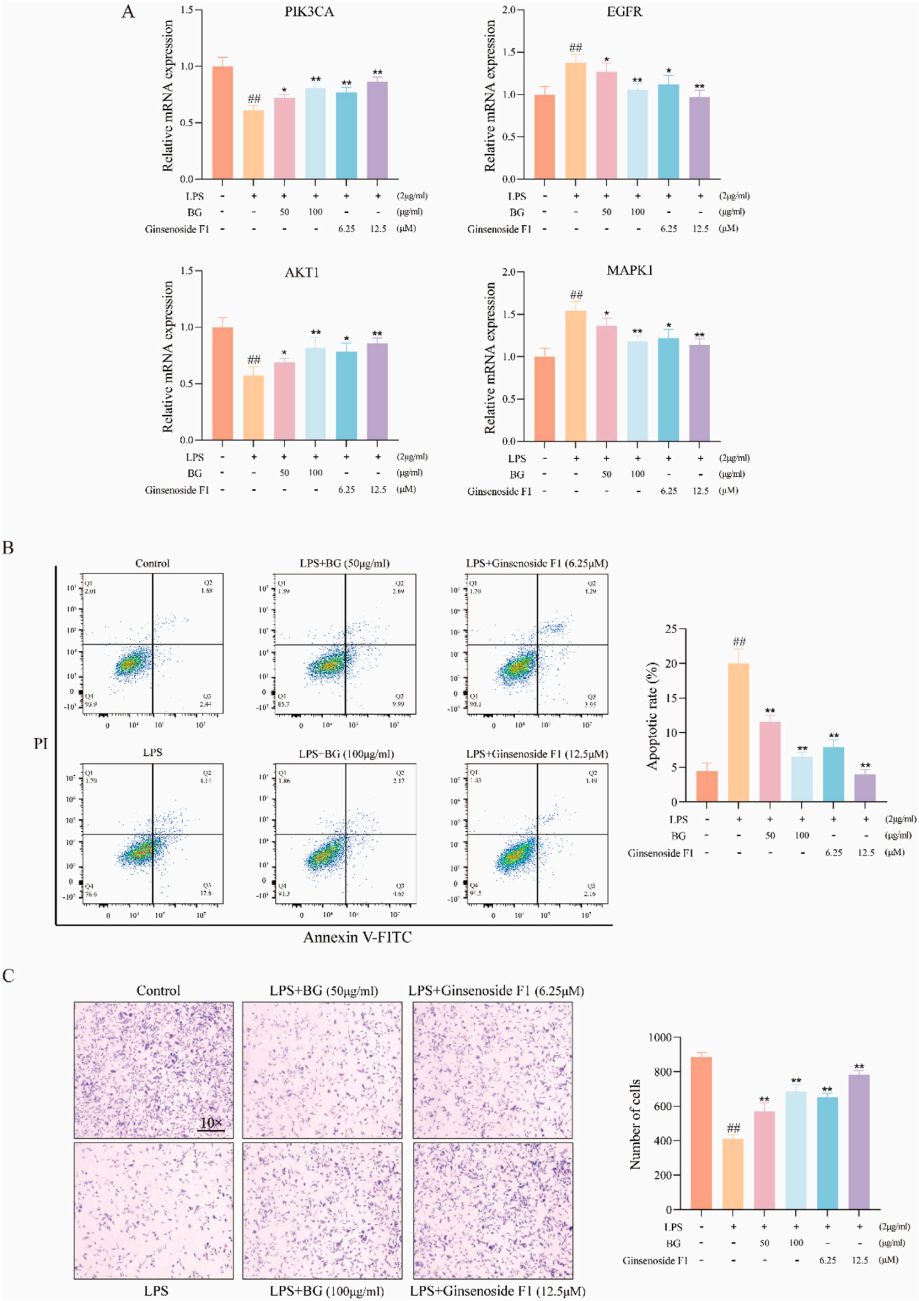
Activity and mechanism verification. **(A)** Relative expression of each gene; **(B)** Apoptosis; **(C)** Cell migration (data expressed as mean ± SD [*n* = 3] ^##^ *p* < 0.01 compared to the control group, *p < 0.05, **p < 0.01 compared to the model group).

Figure 7A demonstrates that ginsenoside F1 and BG can modulate key targets within the PI3K-Akt signaling pathway, including AKT1, MAPK1, PIK3CA, and EGFR. In comparison to the control group, the model group exhibited a significant increase in the mRNA expression levels of MAPK1, PIK3CA, and AKT1, while the mRNA expression level of EGFR was significantly reduced.

### 3.9 Ginsenoside F1 attenuates LPS-induced apoptosis of HT22 cells

The effects of BG and ginsenoside F1 on LPS-induced apoptosis in HT22 cells were assessed using flow cytometry. As depicted in Figure 7B, exposure to 2 μg/mL LPS induced apoptosis in HT22 cells, whereas BG and ginsenoside F1 effectively mitigated LPS-induced apoptosis. The findings indicate that ginsenoside F1 more effectively attenuated LPS-induced apoptosis.

### 3.10 Determination of HT22 cell viability induced by LPS

A Trans well migration assay was employed to evaluate cell viability. The results revealed that, relative to the control group, LPS significantly inhibited the migration of HT22 cells (Figure 7C), while ginsenoside F1 and BG enhanced the viability of HT22 cells compromised by LPS.

## 4 Discussion

Depression is a prevalent mental disorder characterized by persistent sadness, cognitive impairment, and diminished interest, significantly impacting patients’ quality of life (Sampogna et al., 2024). Currently, pharmacological treatment primarily involves Western medicine, though its efficacy is constrained by delayed therapeutic onset and adverse effects, such as headache, weight fluctuations, and cognitive dysfunction (Gill et al., 2020). These limitations underscore the urgent need for safer and more effective antidepressant therapies. Neuroinflammation has been identified as a critical etiological factor in depression, referring to the inflammatory response within the nervous system (Beurel et al., 2020). This term is often used to describe the entirety of immune activities in the central nervous system (CNS) in response to acute and chronic conditions, including trauma, infection, ischemia, autoimmunity, and degeneration. Traumatic brain injury (TBI), neurodegenerative diseases, and other CNS injuries can precipitate neuroinflammation. This process involves a complex immune regulatory mechanism, wherein activated immune cells secrete various pro-inflammatory mediators through positive feedback regulation, including key factors such as tumor necrosis factor-alpha (TNF-α) and interleukin-6 (IL-6) (Amanollahi et al., 2023). Recent scholarly investigations have established a significant association between this pathological process and neurodegenerative disorders, including Alzheimer’s and Parkinson’s diseases, as well as its involvement in the pathogenesis of depression (Gao et al., 2023). This process serves as a common pathological foundation for the onset and progression of these conditions (Lonkar et al., 2025).

Extensive research has demonstrated that ginsenosides exhibit notable therapeutic effects in the treatment of central nervous system disorders (Kim et al., 2013). Specifically, ginsenoside Rd, an active compound found in ginseng, possesses a broad spectrum of pharmacological properties and has been shown to mitigate neuronal damage that can lead to neurological disorders such as depression, Alzheimer’s disease, Parkinson’s disease, cognitive impairment, and cerebral ischemia (Chen et al., 2022). Ginseng has been identified as having potential preventive and therapeutic roles in managing depression (Hou et al., 2020). Furthermore, ginseng and its formulations have been shown to modulate the monoamine neurotransmitter system, enhance the expression of neurotrophic factors, and regulate the function of the hypothalamic-pituitary-adrenal (HPA) axis (Li, 2024). Ginsenoside Rb1 mitigates the aberrant synaptic plasticity in the hippocampus of mice subjected to chronic unpredictable mild stress (CUMS) via the BDNF-TrkB signaling pathway, which is modulated by miR-134, thereby exhibiting an antidepressant-like effect (Wang et al., 2022). Additionally, Ginsenoside Re activates the BDNF/TrkB/ERK/CREB pathway to confer neuroprotection and exerts antidepressant effects by inhibiting oxidative stress and inflammation (Chen et al., 2024). The processed form of BG represents a novel product that generates “rare saponins” and secondary ginsenosides, possessing anti-inflammatory (Huo et al., 2023), anti-cancer (Yu et al., 2025), anti-tumor (Wang et al., 2025), and anti-aging properties (Lee et al., 2018), along with immune regulatory functions (Kim et al., 2019). Furthermore, BG has been reported to alleviate fatigue and depression/anxiety (Lee et al., 2020), demonstrating superior efficacy compared to FG in certain therapeutic contexts. Research by Evelyn Saba et al. indicates that BG brain tonic extract significantly ameliorates scopolamine-induced learning and memory deficits, potentially through mechanisms involving the inhibition of nitric oxide (NO) release, reduction of proinflammatory factor expression, and modulation of the MAPK/NF-κB signaling pathway (Saba et al., 2017). The findings indicate that the extract enriched with BG not only mitigates neuroinflammation but also holds potential as a therapeutic agent for depression.

**FIGURE 8:**
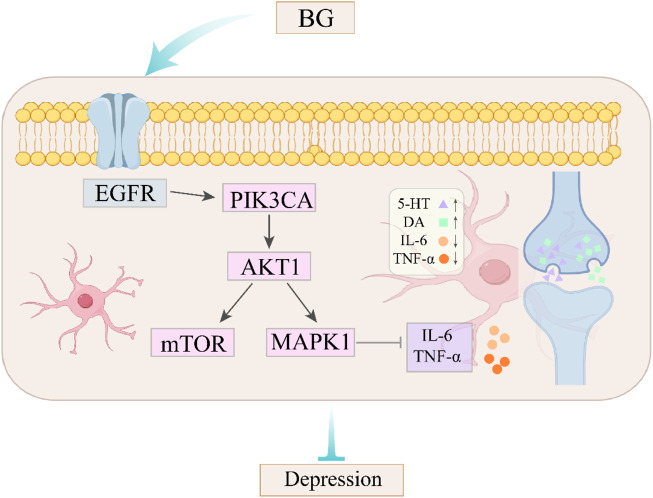
The effect of BG on PI3K/Akt pathway and its relationship with depression were described.

In this study, UPLC-QE Orbitrap-MS technology was employed to qualitatively analyze the chemical constituents of BG, leading to the identification of 25 compounds. Through the analysis of potential active components, network pharmacology was utilized to predict targets, which were then integrated with disease targets to identify 120 key targets. Among these, 18 active components, including rare saponins Rg3 and Rh2, were identified. Further analysis of KEGG pathway enrichment revealed that the targets are primarily concentrated in the PI3K-Akt signaling pathway, involving core targets such as AKT1, MAPK1, PIK3CA, and EGFR. These targets are implicated in the pathological processes of depression by modulating neuroplasticity, anti-apoptosis, and inflammatory responses. In depressive states, there is a reduction in PI3K-Akt activity, leading to the over-activation of GSK-3β and a consequent decrease in the neuroplasticity of the hippocampus and prefrontal cortex (Ludka et al., 2016). This pathway facilitates synaptic protein synthesis, dendritic growth, and neurogenesis by activating the downstream molecule glycogen synthase kinase-3 beta (GSK-3β) (Xian et al., 2019). Akt1 serves as the principal effector within the phosphoinositide 3-kinase (PI3K)-Akt signaling pathway, and the absence of Akt1 kinase activity has been associated with the onset of neurological disorders. Research conducted by Nishisaka et al. demonstrated that chlorogenic acid can activate the Akt1-CREB-RNF146 pathway in the brain, thereby enhancing RNF146 expression and conferring neuroprotection (Kim et al., 2020). Mitogen-activated protein kinase 1 (MAPK1) is involved in the inflammatory response by modulating the nuclear factor kappa-light-chain-enhancer of activated B cells (NF-κB) pathway, which regulates the secretion of interleukin-6 (IL-6) and tumor necrosis factor-alpha (TNF-α), and can mitigate inflammatory reactions and oxidative stress (Cai et al., 2024). Phosphatidylinositol-4,5-bisphosphate 3-kinase catalytic subunit alpha (PIK3CA) is the catalytic subunit of PI3K, responsible for converting phosphatidylinositol 4,5-bisphosphate (PIP2) into phosphatidylinositol 3,4,5-trisphosphate (PIP3) and activating downstream AKT. Studies by Ruolan Sun and others have shown that Bupleurum chinense significantly reduces the expression of phosphorylated PI3K (p-PI3K) protein in the hippocampus of chronic unpredictable mild stress (CUMS) mice, thereby alleviating neuronal damage (Sun et al., 2024). The epidermal growth factor receptor (EGFR) influences the synaptic transmission efficiency of serotonin (5-HT) and dopamine (DA) neurons (Figure 8), and its overexpression may exacerbate depression through inflammatory mechanisms (Zhang et al., 2022).

In this study, the application of advanced UPLC-QE Orbitrap-MS technology, in conjunction with network pharmacology, cellular function assays, molecular dynamics simulations, and PCR analysis, systematically elucidates the antidepressant effects and mechanisms by which BG and its pyrolysis products inhibit neuroinflammation. The study accurately identified saponins and their pyrolytic derivatives, such as the rare saponins Rg3 and Rh2, within BG, thereby establishing a reliable material basis for further research. Utilizing an HT22 cell model of neuroinflammation induced by LPS, ginsenoside F1 was identified as the key active component in BG that ameliorates neuroinflammation. Molecular docking results demonstrate that the core targets within the PI3K-Akt signaling pathway (AKT1, MAPK1, PIK3CA, EGFR) exhibit strong affinity with the key active components. Furthermore, molecular dynamics simulations confirm that these components and proteins form stable conformations. PCR analysis corroborated that BG and ginsenoside F1 significantly influence the expression levels of these targets, providing robust support for the network pharmacology findings. The effects of BG and ginsenoside F1 on cell apoptosis and viability were confirmed through cell flow cytometry and migration assays. The findings indicate that the active components of BG exert a significant regulatory effect on key targets. It is hypothesized that BG mitigates neuroinflammation by modulating the PI3K-Akt, MAPK, and other inflammatory pathways via rare saponins, such as ginsenoside F1, thereby contributing to the treatment of depression. Nonetheless, this study has certain limitations, and future research should involve *in vivo* animal models to conduct more comprehensive investigations, thereby providing more robust data on the efficacy of BG in treating depression.

Overall, this study not only proposes a novel strategy for addressing neuroinflammation-related disorders but also lays a solid foundation for the use of ginsenoside F1 in depression treatment and supports the clinical application and drug development of BG.

## 5 Conclusion

In conclusion, this study aims to comprehensively investigate the active compounds and mechanisms of BG in combating neuroinflammation, focusing on component pyrolysis. Utilizing UPLC-QE Orbitrap-MS in conjunction with network pharmacology, the research examines the antidepressant properties of BG. The analysis identified 25 potential active ingredients, with ginsenoside F1 emerging as the primary active compound through cell-based experiments. Molecular dynamics simulations and additional methodologies demonstrated that both BG and ginsenoside F1 exhibit stable and sustained activity, effectively inhibiting neuroinflammation and exerting antidepressant effects. It is hypothesized that BG may inhibit key targets within the PI3K-Akt signaling pathway and others, thereby mitigating neuroinflammation and reducing cell apoptosis. Consequently, BG presents itself as a promising natural therapeutic agent for the treatment of depression.

## Data Availability

The original contributions presented in the study are included in the article/supplementary material, further inquiries can be directed to the corresponding authors.
